# Latent class analysis and longitudinal development trajectory study of psychological distress in patients with stroke: a study protocol

**DOI:** 10.3389/fpsyt.2024.1326988

**Published:** 2024-06-03

**Authors:** Yunmei Guo, Ming Zhou, Xin Yan, Ying Liu, Lianhong Wang

**Affiliations:** ^1^ Nursing Department, Affiliated Hospital of Zunyi Medical University, Zunyi, Guizhou, China; ^2^ School of Forensic Medicine, Zunyi Medical University, Zunyi, Guizhou, China

**Keywords:** latent class analysis, psychological distress, stroke, LCA, LTA

## Abstract

**Background:**

Psychological distress affects the treatment and rehabilitation of patients with stroke, affects their long-term functional exercise and quality of life, and increases the risk of stroke recurrence and even death. This is a multi-dimensional and multi-level mental health problem and a dynamic process variable that shows a dynamic development trend with time. However, previous studies have been insufficient to deeply study the change mechanism of psychological distress, and there remains a lack of forward-looking longitudinal studies to analyze its change trajectory. This study aimed to investigate potential categories and how psychological distress changes over time and to examine conversion probability in these transformation processes.

**Methods:**

This prospective longitudinal mixed-method study investigated the potential categories and change trajectories of distress in patients with stroke. A total of 492 participants from three hospitals were recruited for quantitative analysis. Latent class analysis and latent transition analysis (LCA/LTA) were used to identify meaningful subgroups, transitions between those classes across time, and baseline demographic features that help predict and design tailored interventions.

**Discussion:**

A comprehensive understanding of the potential category and transformation processes of psychological distress over time, including the impact of the sense of demographic data on the role of shame and loneliness, can lead to the development of psychological distress treatment tailored to the unique needs of patients with stroke. Thus, this study can promote more effective and successful treatment outcomes, reduce the stigma surrounding disease issues among patients, and encourage them to use psychological consultation.

## Background

Stroke is characterized by high morbidity, disability, mortality, and recurrence and is the primary cause of death and disability in adult men and women worldwide (1). The 2016 Global Burden of Disease data show that stroke is the leading cause of years of life lost in China (2), indicating that stroke is a great challenge. The incidence and prevalence of stroke have recently increased (3, 4). Approximately 60%–80% of patients with stroke have different degrees of dysfunction in post-stroke treatment and rehabilitation and may have great psychological distress due to disability and self-image disorder (3, 4). Studies have shown that about one-third of stroke survivors in Western developed countries experience psychological problems soon after their illness (5). Psychological distress impedes the treatment and rehabilitation of patients with stroke and affects their long-term functional exercise and quality of life, increasing the risk of stroke recurrence and death (6).

Psychological distress, also known as emotional distress, psychological stress, and psychological pain, is a common mental health problem. This unpleasant emotional experience is caused by multiple factors. It is a sub-health state between normal psychology and mental illness, often involving psychological, social, and spiritual levels, including depression, anxiety, fear, compulsion, and paranoia (7). The social level includes social problems, such as hostility, isolation, and interpersonal sensitivity (8), while the mental level includes somatization symptoms, a sense of shame, and role maladjustment. However, most related studies on the psychological distress of stroke patients are based on a single level of research, with post-stroke depression as the psychological distress of patients with stroke. However, Crowe et al. (9) found that psychological distress in patients with stroke mainly manifests in three major themes: fear, self-loss, and loneliness. A longitudinal (10) study in China also showed that psychological distress mainly manifests as anxiety and depression, as well as hostility, indifference, anger, depression, and poor interpersonal relationships. Therefore, it is necessary to break through the existing single level to focus on the psychological bottlenecks of patients with stroke.

Additionally, stroke has a unique disease trajectory that differs from that of other chronic diseases, deteriorating periodically with the acute onset of the disease, resulting in a sharp decline in body function. This highly uncertain trajectory emphasizes the urgent need for a longitudinal study of patients with stroke, as psychological distress has dynamic characteristics that cannot be captured at a single time point (11). However, few longitudinal studies have been conducted on the psychological distress of stroke patients at home and abroad, and none have classified the psychological pain of patients with stroke and tracked their dynamic changes. Therefore, it is of great significance to analyze the potential categories and developmental trajectory of psychological distress in patients with stroke in multiple dimensions.

Latent class analysis (LCA) is a multivariate statistical model that can estimate the probability of each participant assigned to each subgroup according to the maximum likelihood and assigns each participant to the group to which they belong with the highest chance (12, 13). This method can focus on the heterogeneity of psychological distress in patients with stroke and divide psychological distress into subtypes that reflect certain characteristics, which can describe the quantitative differences between individuals and summarize the qualitative differences between classes or groups. Recently, LCA has been used in medicine, sociology, psychology, and other fields (14–16), presenting that it is an effective and practical method. Currently, there are no studies at home and abroad using LCA to analyze the psychological distress of patients with stroke to pay attention to the heterogeneity and diversity of their psychological distress.

Latent transition analysis (LTA) is a longitudinal extension model of LCA that extends the analysis framework from a single time-point analysis to a longitudinal data analysis in which the same individual is measured at two or more time points (17). LTA uses the transition matrix to describe the stability of the individual by describing the invariant probability of the potential category to which the individual belongs and describes the development trend from the transition probability to obtain the development trajectory. After adding covariates, we obtained a correlation analysis of the relevant transformation factors. We can further explore the correlation analysis of various influencing factors in different types of psychological distress and their transformation from the perspective of time development. Then, we obtained the developmental track of psychological distress and its related factors in patients with stroke. However, no study has analyzed the potential categories of psychological distress in patients with stroke using LCA and LTA to investigate the longitudinal trajectory of patients with stroke to pay attention to its dynamic development trend over time.

Therefore, this study aimed to identify the psychological distress of patients with multi-dimensional (psychological–social–mental), break through the bottleneck of the existing research on identifying psychological distress at a single level, classify the psychological distress of patients with stroke using LCA, and dynamically identify the dynamic trajectory of psychological distress in patients with stroke. This study’s findings may provide a reference basis for psychological intervention of patients with this disease.

## Methods

This was a prospective, longitudinal study. Baseline data were collected when the patients’ condition had stabilized. Follow-up (WeChat telephone or face-to-face) studies were conducted at 3 months, 6 months, 9 months, and 12 months after onset. The eligibility criteria are presented in **Table 1**.

**Table 1:** Elegibility criteria.

Inclusion criteria
(1) Patients with acute ischemic stroke diagnosed by MRI in accordance with the diagnostic criteria of “Clinical diagnosis and treatment Guide Neurology Branch” compiled by Chinese Medical Association in 2010;
(2) Patients with stable condition, stable vital signs, clear consciousness and no aphasia after medical treatment;
(3) 18-64 years old;
(4) Patients with informed consent before the study.
Exclusion criteria
(1) Heart, liver, kidney and other organs are seriously dysfunctional and have malignant tumors;
(2) Severe aphasia and cognitive dysfunction;
(3) Participating in other research projects.

### Aims

**Figure 1:**
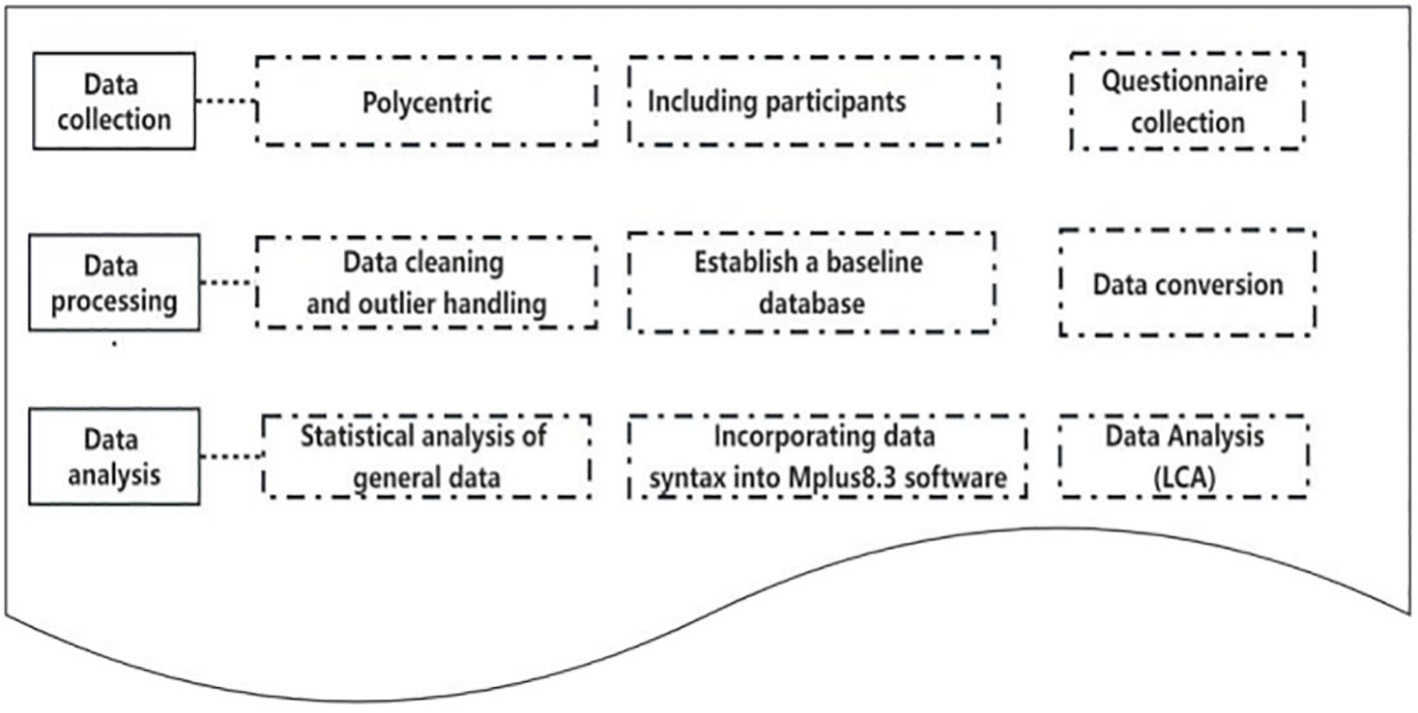
Lacent class analysis and classification characteristics of psychological disturbance in patients with stroke.

This study primarily aimed to classify the psychological distress of patients with stroke using LCA and dynamically identify the dynamic track of psychological disturbances in patients with stroke. The specific aim was to examine the following:

Specific Aim 1: Potential classification and characteristics of psychological disorders in patients with stroke (**Figure 1**).

**Figure 2:**
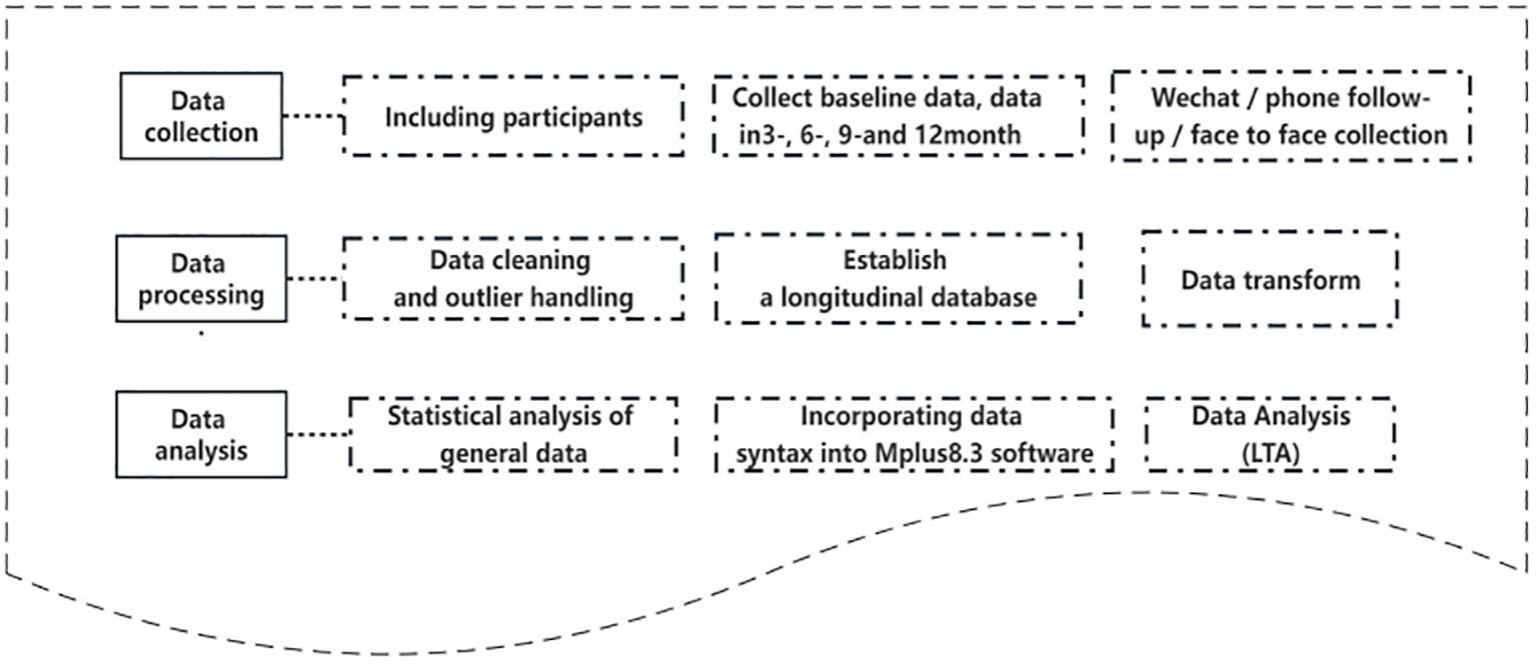
Development track and correlation analysis of psychological disturbance in patient with stroke.

Specific Aim 2: Development of track and correlation analysis of psychological disturbances in patients with stroke (**Figure 2**).

### Study design and population

**Figure 3:**
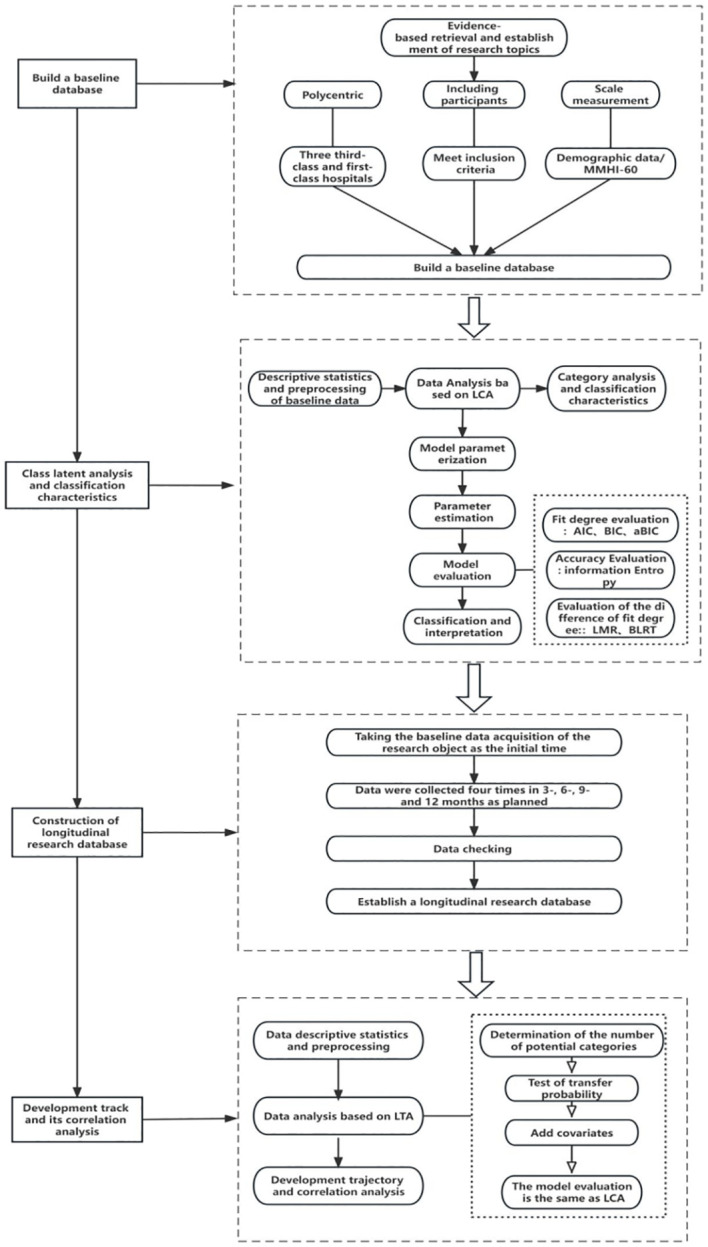
Analysis of potential categories of psychological distress and roadmap of longitudinal changes in patients with stroke.

This was a prospective longitudinal study in which patients with stroke were recruited by convenience sampling from three tertiary hospitals in Zunyi City, Guizhou Province, China. This study was conducted from January 2024 to June 2025. The Strengthening the Reporting of Observational Studies in Epidemiology (STROBE) and reporting of studies conducted using routinely collected data (RECORD) Statements were followed in reporting the complete research (**Figure 3**) (18).

### Determination of the sample size

This study’s sample was mainly recruited from April to October 2024 at the affiliated Hospital of Zunyi Medical University, which met the criteria for stroke and had a minimum standard sample size of LCA ≥ 500 (19).

### Data collection procedure

Two uniformly trained researchers identified potential subjects in the outpatient clinic and established the final issues according to the inclusion and exclusion criteria. First, a researcher guided the subjects to a quiet classroom in batches to facilitate conversation and data collection. Another researcher adopted a one-to-one form to introduce and explain the study’s primary purpose, significance, and implementation process; promised the principle of privacy protection; and acquired signed informed consent forms after obtaining the willingness of the research subjects to participate. After completing the baseline data, the researchers conducted four follow-up surveys and instructed the participants to fill in the surveys face-to-face, by phone or WeChat, or in the clinic at the corresponding point in time. After collecting data from the research subjects, the data were saved in the background and extracted into an Excel file. When all the data were collected, they were extracted and input independently by two researchers and cross-checked. If there were differences, they were resolved through negotiation. After collecting data, LCA processing and LTA model construction were conducted.

### Quality control

1. The subjects were selected strictly according to the inclusion and exclusion criteria, and they signed an informed consent form before completing the questionnaire.
2. Investigator training: The researchers were trained before the formal investigation, and unified standards and survey methods were conducted to ensure the objectivity and accuracy of the survey data.
3. Quality control to reduce the loss of follow-up rate: Based on the study subject’s baseline data collection time, the researchers were notified and reminded via WeChat or telephone 1 week before each subsequent data collection. On the same day as the survey, the researchers contacted the subjects to remind them that they had to complete the questionnaire. After completing the questionnaire, recovery efficiency was ensured by giving the participants small gifts.
4. Quality control of the survey: Before the first survey, the contents of each scale and matters needing attention were explained to ensure the authenticity and accuracy of the survey results. Uniform terms were used to describe the items they did not understand, avoiding induced language. The questionnaire must be filled out and checked immediately after completion, and it must be verified and corrected if necessary.
5. Survey data quality control: The obtained data were double-entered and checked. The original data were checked and corrected in time if there were any differences to ensure the data accuracy.
6. Quality control of model construction: Explicit variables were measured repeatedly at multiple time points in the LTA. The concatenated list could be extensive, possibly leading to sparse data due to the small ratio of sample size to the number of attached table cells. Thus, the number of subjects analyzing some answer patterns was zero, making it difficult for model selection, parameter estimation, and logistic regression. This problem can be solved by restricting the effective parameters and using the BETAPROR statement in the program to set an *a priori* distribution. Simultaneously, manually checking the conditional probabilities of different potential states can eliminate the problem of possible state order caused by label transition.

### Measures

#### General demographic data

General demographic data of the subjects, including name, age, education level, and monthly income of the family, were collected by the researchers themselves.

#### Symptom Checklist-90 

The Symptom Checklist-90 (SCL-90) scale was used to measure psychological disturbances in the patients (20). The scale consists of 90 items, including somatization, obsessive-compulsive symptoms, interpersonal sensitivity, depression, anxiety, hostility, phobia, paranoia, psychosis, and nine other factors. Cronbach’s α coefficient of the scale was 0.98. According to the 5-point scoring method, 0–4 corresponds to “no”, “very light”, “moderate”, “heavy”, and “serious”, respectively, and the total score reaches 160 or any factor score reaches 2.

#### Social impact scale 

Patients’ sense of shame was measured using this scale (21). The scale consists of 24 items and four dimensions: social exclusion (nine items, 1–9), economic insecurity (three items, 10–12), inherent shame (five items, 13–17), and social isolation (seven items, 18–24). Cronbach’s α coefficient for the scale was 0.828. All entries were scored on a 4-point Likert scale, with 1–4 corresponding to “extremely disagree”, “disagree”, “agree”, and “highly agree”, respectively. The total score was 240.96. The higher the score, the stronger the sense of shame. The median score on the scale was 48 as the dividing line to determine whether there was a sense of guilt, 48 as no shame, and ≥48 as a sense of shame.

#### University of California at Los Angeles—Loneliness scale 

The loneliness of patients was measured using the University of California at Los Angeles (UCLA) scale (22). This scale was compiled by Russell et al. The internal consistency and test–retest reliability of the scale were 0.89. There were 20 items, including 11 positive-order items (2, 3, 4, 7, 8, 11, 12, 13, 14, 17, and 18 items) and nine reverse-order items (1, 5, 6, 9, 10, 15, 19, and 20 items). The positive-order items (never, rarely, sometimes, and always) were scored 1–4, and the reverse-order items (never, rarely, sometimes, and always) were scored 4–1. The higher the score, the higher the loneliness of the subjects. The total score on the scale was 20–80; a score of 30–34 indicated a low degree of loneliness, a score of 35–49 indicated moderate loneliness, and a score of 50–80 showed a higher degree of loneliness.

#### Self-efficacy scale 

Self-efficacy (SE) was assessed using the General Self-Efficacy Scale, comprising 10 items (23). A 4-point Likert scale was used to answer each question, ranging from 1 (not at all true) to 4 (exactly true). Total scores ranged from 10 to 40, with higher scores indicating better SE. The internal consistency of the SE scale was good, with a Cronbach’s α of 0.96.

#### Health literacy scale 

In this study, the Chinese version of the Health Literacy Management Scale (HeLMS) (24) was used to evaluate health literacy (HL) in the patients. This questionnaire consists of 24 items in four dimensions: communication and interaction ability (nine items with a total score of 45 points), information acquisition ability (nine items with a total score of 45 points), willingness to improve health (four items with a total score of 20 points), and willingness to support health financially (two items with a total score of 10 points).

Each item is rated on a 5-point Likert scale ranging from 1 (very difficult) to 5 (not at all difficult), with higher scores indicating a more advanced level of HL. The total score was 120 points. An average score of <4 on all scale dimensions was considered inadequate or low level of HL. The internal consistency of the HeLMS scale was good, with a Cronbach’s α of 0.874.

### Covariates

This study examined patient demographics, including gender, race/ethnicity, age, primary insurance type, quality of life, self-efficacy, health literacy, and patients’ sense of shame, as potential predictors of transitions over time.

### Promotion of study participation

Participants were contacted and informed 2 weeks before the next quantitative survey was conducted. Participants had access to data for the questionnaire via WeChat or phone. For each survey, the participants received a remuneration of 10 and the same reward during a follow-up of four times over 12 months.

### Data analysis plan

The general data of patients were described, analyzed, and preprocessed using SPSS 18.0 software. This primarily included data cleaning, abnormal value processing, and conversion, with statistically significant differences (p < 0.05).

The statistical method used Mplus8.3 to analyze the potential categories. First, the data measured by the psychological distress scale and each dimension item were converted into 0 and 1. The total score of the original item of the psychological distress scale was ≥160, or the average score of any sub-dimension measurement table was ≥2 as a positive item, and the assignment was 1. The total score of the original item was <160, or the average score of any sub-dimension measurement table was <2 as a negative item, and the assignment was 0. The process included model parametric parameter estimation, model evaluation, potential classification, and result interpretation. The fitting process started from the initial model, assuming only one category in all samples. The number of classes in the model was gradually increased until the model with the best-fitting data was obtained.

The main evaluation indices of the LCA model included the Akaike information criterion (AIC), Bayesian information criterion (BIC), sample-corrected Bayesian information criterion (aBIC), average information index (Entropy), likelihood ratio test [Lo–Mendell–Rubin (LMR)], and bootstrap likelihood ratio test bootstrap (BLRT). Among them, the smaller the values of AIC, BIC, and aBIC, the better the model fitting. Entropy is an index to evaluate the accuracy of classification. LMR and BLRT were used to compare the relevant differences between k-mai-1 and k-category models. The p-value of the two models reached a significant level, indicating that the k-category model was better than the k-Mel one-category model.

Then, the longitudinal research database data were based on LCA using the typical method deviation test, which included determining the number of potential categories and the transfer probability test and adding covariables to obtain the development trajectory and association analysis. The Harman single-factor method was used to test the standard method deviation, and unrotated principal component factor analysis was conducted on the data of all measurement items at four time points. More than one common factor was extracted from the four time points, and the interpretation rate of the first common factor was much less than the critical standard of 40% for judging whether there was an apparent standard deviation in this study.

After passing the common variance test, the number of potential categories without adding any covariables was determined to obtain the potential transformation model and analyze the LTA transformation rate. AIC, BIC, and aBIC were used to evaluate the LTA model fitting; Entropy was used to evaluate the classification accuracy; LMR and BLRT were used to compare differences. The evaluation criteria for the above indicators were the same as those for LCA. Additionally, when the number of categories increased but we could not find the best model, we used a steep slope graph to determine the optimal number of classes when the statistical index decreases sequentially according to the obvious inflection point. Finally, taking the demographic characteristics of psychological disturbance and disease characteristics as independent variables and the type of LCA transformation as dependent variables, the primary multivariate logistic regression method and Mplus8.3 software were used to analyze the correlation between the factors affecting the potential transition probability.

### Ethics and timeline

**Figure 4:**
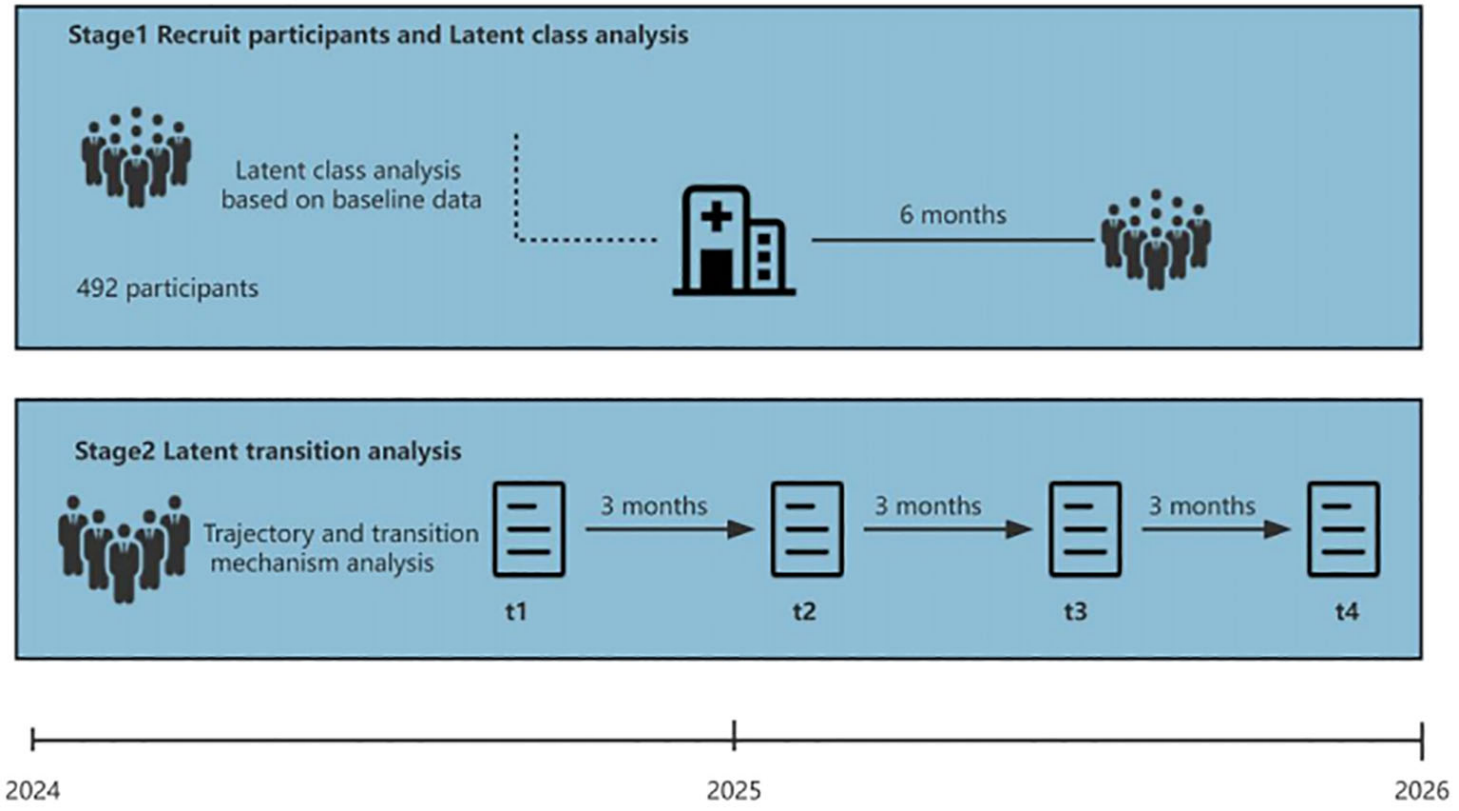
Participant timeline.

This study involved human participants and was approved by the Ethical Committee of the Affiliated Hospital of Zunyi Medical University (No. KLL-2023-533). The participants provided informed consent to participate before the start of the study. The time points for the two stages are displayed in **Figure 4**.

## Discussion

Although most studies have shown that psychological distress plays a vital role in the indicators of health outcomes of convalescent patients after stroke, previous studies were mainly based on a single-level survey and did not analyze the potential categories of psychological distress. Simultaneously, psychological distress is a dynamic process variable that shows a dynamic development trend with time. However, previous studies have not profoundly studied the changing mechanism of psychological distress, and there remains a lack of forward-looking longitudinal studies to analyze changing trajectories.

This study addresses several research gaps. First, the exploratory LCA method was used to explore the group heterogeneity in the psychological disturbance status of stroke patients, examine the population characteristics of different psychological disturbance subgroups, and provide a basis for formulating and implementing individualized intervention measures for stroke patients with varying factors in the future. Second, a longitudinal study was used to track the possible changes in psychological distress in stroke patients over time and to reveal the probability of conversion among potential categories of psychological distress in this population and combined with multi-valued logistic regression analysis to explore the factors affecting the likely category conversion probability of stroke patients to provide a theoretical and practical basis for expanding the means and content of clinically accurate interventions. Finally, the mixed-method approach led to a more comprehensive understanding of the transformation processes by combining robust statistical data on latent classes with deep insights into subjective perspectives and contextual factors.

Multi-dimensional (psychological–social–mental) identification of patients’ psychological distress included breaking through the bottleneck of existing research on identifying psychological distress at a single level and classifying the psychological distress of stroke patients by potential category analysis. The panoramic analysis of possible transformation analysis was used to dynamically identify the dynamic trajectory of patients’ psychological distress to provide a reference basis for psychological disturbance interventions in patients with this kind of disease. The smooth development of this study may provide a new perspective for formulating accurate target strategies for psychological intervention in stroke patients, further helping reduce their sense of shame and encouraging them to use mental health services.

## Ethics statement

The studies involving humans were approved by Affiliated Hospital of Zunyi Medical University (No. KLL-2023-533). The studies were conducted in accordance with the local legislation and institutional requirements. Written informed consent for participation in this study was provided by the participants’ legal guardians/next of kin.

## Author contributions

YG: Investigation, Software, Writing – review & editing. MZ: Data curation, Software, Writing – review & editing. XY: Conceptualization, Writing – original draft. YL: Formal analysis, Software, Validation, Writing – original draft. LW: Conceptualization, Software, Supervision, Writing – original draft, Writing – review & editing.
